# Supplementary material for: Birth month as a risk factor of allergic diseases: Analysis of database of about 50 thousand children
Source: PLoS One. 2025 Nov 20;20(11):e0336062. doi: 10.1371/journal.pone.0336062 (PMC12633921; doi:10.1371/journal.pone.0336062)
Supplement: Attachment — Submitted filename: Response to Reviewers.docx [file pone.0336062.s001.docx]

Response to Reviewers:

**Reviewer #1**

1. The conclusion implies causality (e.g., "seasonality is an independent factor"). This wording should be revised to reflect an association rather than causation due to the observational nature of the study.

The authors' response:

Dear reviewer! We have changed the wording (e.g., "seasonality is an independent factor") to reflect an association rather than causation.

2. While asthma was included, its adjusted odds ratios were not statistically significant. This point should be addressed more clearly in the Discussion section.

The authors' response:

Dear reviewer! The absence of a statistically significant association between asthma and month of birth is noted in the Discussion section.

3. Limitations such as parental recall bias, self-selection in the data collection process, and potential unmeasured confounding (e.g., vitamin D levels, exposure to specific allergens) should be more

explicitly discussed.

The authors' response:

Dear reviewer! We have included additional points in the limitation section.

4. Figures and tables should include more descriptive legends for better standalone comprehension.

The authors' response:

Dear reviewer! We have corrected the title of table 2.

5. Minor language edits are suggested, such as:

“requiring merits further investigation” → “which merits further investigation”

"In the Russian" → "In Russia"

The authors' response:

Dear reviewer! We have corrected minor language edits.

**Reviewer #2**

1. METHODS: The main issue with this study is that it potentially is affected by selection bias. The study population has been selected as children who went to a pediatric visit. Does this mean that the study population is only children who have some kind of illness or do all children in Russia go to pediatricians? I think more context needs to be supplied. In Western countries usually only children with very particular issues go to pediatricians, and often only if their parents can afford it. Therefore, there could be two layers of selection bias which effect the generalisability of these findings - only children with illnesses, and only wealthy people. Please put more context and description around these points. The fact that there are 50 000 children who go to pediatricians in only a 9 month period seems really large, so I think explanations about practices in Russia would help. Is there a way to include the general population in this study? if not, then it needs to be specified in the abstract, main findings, discussion that these findings are within a population of children with health issues and the issues with generalisability must be discussed in the Limitations section.

The authors' response:

Dear reviewer! In Russia, the basic medical care for children and adults is provided through compulsory health insurance, i.e. free of charge. Children may visit a pediatrician due to illness (e.g., often in the case of an acute viral infection or in connection with some specific complaints or diseases), or patients may come without complaints or diseases, e.g., to obtain school or sectional certificates, to undergo a checkup or vaccination. Therefore, the study population included both children with diseases and healthy children. In our study there are no such two levels of selection bias: children with diseases and only wealthy people. We have added additional information to the Methods section.

2. METHODS: Is there a way the authors can adjust for socioeconomic status which is an important confounder and potential source of selection bias in this study? if not, please discuss in limitations and its effect.

The authors' response:

Dear reviewer! Visiting a pediatrician in Russia is free of charge, so socioeconomic status is not an important factor and potential source of selection bias in this study. The EMIAS system does not take into account the socioeconomic status of the family. We have included this aspect in the limitation section.

3. METHODS: Is it possible to adjust for month of conception or adverse perinatal outcomes such as gestational age? these could both be affecting the outcomes.

The authors' response:

Dear reviewer! Unfortunately, we cannot make an adjustment for the month of conception and gestational age, since this information is not in the EMIAS system. We have included this aspect in the limitation section.

4. METHODS: Related to point 1, please provide more information about what the EMIAS is and how data is collected.

The authors' response:

Dear reviewer! We have added information about EMIAS to the Methods section.

5. METHODS: Was it the pediatricians who assigned the ICD-10 code from the visit?

The authors' response:

Dear reviewer! If a child visits a pediatrician with a disease, the pediatrician makes a diagnosis with diagnosis code according to ICD-10.

6. METHODS: I think it would help if you provided inclusion and exclusion criteria to the study population. I am unsure if they only people included were those with allergy or whether the ICD 10 codes were used to define the outcomes of interest.

The authors' response:

Dear reviewer! The main inclusion criteria for the formation of the database from EMIAS were: age under 18 years and a parentally completed questionnaire to identify family allergy history. We have specified these criteria in the methods section.

7. RESULTS: I am surprised that the children started at age 4 years, since AD, AR and asthma often show up in younger children. Please again provide context as to why this is.

The authors' response:

Dear reviewer! The median age of the children whose parents completed the questionnaire is given in the article. This age is not related to the onset of allergic disease, i.e. the data download only recorded the fact that the child had the disease in the medical record, which could have been at any year of life.

8. RESULTS: The term 'odds' should be used instead of 'risk' eg in the abstract and in the results. Risk implies relative risk which is different to an odds ratio. Similarly use 'association' rather than 'correlation'.

The authors' response:

Dear reviewer! We have changed the term 'risk' to 'odds', and the term 'correlation' to 'association'.

9. RESULTS: Please refer to the formatting instructions of the journal for decimal places and inclusion of p values. Usual practice is to have 2 rather than 3 DP and to not include p values.

The authors' response:

Dear reviewer! We checked the Submission Guidelines (<https://journals.plos.org/plosone/s/submission-guidelines> ) and found the following phrase in the Statistical Reporting section:

“P-values. Report exact p-values for all values greater than or equal to 0.001. P-values less than 0.001 may be expressed as p < 0.001, or as exponentials in studies of genetic associations.”

The practice of reporting p-values with three decimal places while presenting results in medicine has been highlighted in many statistical guidebooks and articles (<https://pmc.ncbi.nlm.nih.gov/articles/PMC7642026/> , <https://pmc.ncbi.nlm.nih.gov/articles/PMC8024217/> , <https://pmc.ncbi.nlm.nih.gov/articles/PMC8005799/> )

Additionally, the third decimal place can be very important, especially in case of adjustment for multiplicity, as can be seen from our tables (for example, p-values of 0.028 or 0.027 from Table 3, that are very close to the adjusted threshold). We would like to present our results to readers in the most transparent way.

10. DISCUSSION: The first paragraph of the discussion should be a summary of the findings, please add this.

The authors' response:

Dear reviewer! We have added the first paragraph to the discussion section.

11. DISCUSSION: there needs to be more discussion about why there is heterogeneity between findings in this area, it is not enough to just say there is heterogeneity, some thoughts on why this is, and what your study adds are needed

The authors' response:

Dear reviewer! Overall, our data and the data from various studies regarding the association of season of birth with the odds of developing AR and asthma demonstrate heterogeneity, but no study showed an increased odds of AR and asthma among those born in the summer months. This observation probably supports the positive effect of warm climatic conditions among those born in the summer months on reducing the odds of developing allergic diseases.

Available data on the effect of the season of birth on the odds of allergy in different regions can vary considerably, demonstrating specific geographic patterns, even within the same country, in addition, the size of the patient sample, research methodology, and adjustments for various factors may be of significant importance for these differences.

We have added this information to the discussion section.

12. DISCUSSION: an important undiscussed factor to explain the associations is perinatal factors associated with season which are also highly related to allergic outcomes. For instance, I am wondering if preterm births, low birth weight and caesarean sections are more likely to happen in winter which could explain the findings?

The authors' response:

Dear reviewer! Cesarean section is an important known and common perinatal factor that most studies suggest may increase the odds of allergic diseases. It could be assumed that the higher frequency of cesarean section in the fall and winter months could to some extent determine the association of the season of birth and the odds of AD and AR that we observed. However, in Russia, cesarean sections are performed only when strict indications exist and there are no data on the frequency of cesarean sections in different periods of the year. In an Iranian study, Nasiri R. et al. reported that seasonal (monthly) variations of the weather (humidity and temperature) have a significant impression on preeclampsia prevalence, which is one of the important indications for cesarean section. Based on time of conception the lowest prevalence of preeclampsia was found in winter and early spring, which corresponds to the lowest frequency of cesarean section in the fall and winter period. Thus, the effect of cesarean section on the association of season of birth and risk of allergic disease is ambiguous but extremely interesting and requires further study.

We have added this information to the discussion section.

13. DISCUSSION: related, there needs to be more discussion about why the month of BIRTH is driving long term outcomes. that it could be affecting outcomes 6 months later as in some other studies makes sense, but month of birth on disease 4-18 years later seems a big claim. How do we know it is not the exposure in the first few months after birth or the month of conception or the exposures during pregnancy? I am trying to suggest to consider in the discussion what month of birth represents in a broader context. As the authors say, there are so many factors that could be influencing (confounding or mediating) these findings. Could the authors suggest given the breadth of literature on this topic which areas the research should branch into rather than more of the same?

The authors' response:

Dear reviewer! Further epidemiologic studies are needed to accumulate evidence of the identified association of season and month of birth with allergic disease odds, and additional research is needed to find new potential factors that may determine and/or strengthen this association. Such factors include, for example, increasing exposure and sensitization to epidermal allergens (cats, dogs) in autumn and winter, viral infections in the first year of life, such as COVID-19, the presence of children older than 2 years of age in the family (i.e., preschool and school age), which contribute to the infant's early exposure to respiratory viruses, the socioeconomic status of the family, which to some extent may offset already known factors (medication correction of vitamin D levels, travel to countries with warmer climates, monitoring of the child's health, etc.), conception season, use of in vitro fertilization, premature birth, negative perinatal factors (prematurity, hypoxia, etc.). In addition, it is relevant to study not only the association of season and month of birth with the risk of allergic disease, but also the possible influence of season of birth on the severity and prognosis of the disease.

We have added this information to the discussion section.
