# Supplementary material for: Birth month as a risk factor of allergic diseases: Analysis of database of about 50 thousand children
Source: PLoS One. 2025 Nov 20;20(11):e0336062. doi: 10.1371/journal.pone.0336062 (PMC12633921; doi:10.1371/journal.pone.0336062)
Supplement: Attachment — Submitted filename: renamed_4ea54.docx [file pone.0336062.s002.docx]

**Reviewer’s Comments**

**To the authors,**

This study makes an important contribution to understanding seasonal birth effects on allergic diseases in Russian children. The large sample size, appropriate statistical methods, and novel geographic context are significant strengths. The findings generally align with global patterns showing increased allergy risk in autumn/winter births, likely mediated by vitamin D deficiency and other seasonal factors. Many methodological and statistical issues have been adequately addressed in previous reviews. I would like to add the following recommendations for improvement:

1. Abstract: Minor spelling corrections
   I. "Crude odd ratio" should be "crude odds ratio" (line 31)
   II. Consider stating the study period (2024) in methods
   III. The phrase "elevated odd" (line 41) should be "elevated odds"
2. Introduction:
I. Lines 58-65: The sentence beginning with "Notably, the season factor itself is heterogeneous..." is overly complex and should be broken into shorter, clearer statements for better readability.
II. The connection between seasonal factors and specific mechanisms (vitamin D pathways, allergen exposure) could be included with more current evidence.
3. Methods: The manuscript does not address how missing data were managed. Authors should either: Describe the specific approach used for handling missing values (e.g., complete case analysis, multiple imputation) OR explicitly state if there were no missing values in the dataset
4. Results: The relatively low asthma prevalence (4.8%) compared to global ISAAC estimates (11.8-13.8%) requires more discussion.
5. Discussion: Recommended additional discussion of potential prevention strategies based on findings
6. Limitations: Missing discussion about generalizability beyond Moscow
7. Conclusion: Recommended to include clinical implications discussion for prevention strategies
