# Supplementary material for: Birth month as a risk factor of allergic diseases: Analysis of database of about 50 thousand children
Source: PLoS One. 2025 Nov 20;20(11):e0336062. doi: 10.1371/journal.pone.0336062 (PMC12633921; doi:10.1371/journal.pone.0336062)
Supplement: Attachment — Submitted filename: Response_to_Reviewers_auresp_2.docx [file pone.0336062.s003.docx]

Response to Reviewer:

1. Abstract: Minor spelling corrections
   I. "Crude odd ratio" should be "crude odds ratio" (line 31)

The authors' response:

Dear reviewer! We have corrected minor spelling edits. (line 31)

II. Consider stating the study period (2024) in methods

The authors' response:

Dear reviewer! The study period (2024) is included in methods. (line 27, line 79)

III. The phrase "elevated odd" (line 41) should be "elevated odds"

The authors' response:

Dear reviewer! We have corrected minor spelling edits. (line 41)

2. Introduction:
I. Lines 58-65: The sentence beginning with "Notably, the season factor itself is heterogeneous..." is overly complex and should be broken into shorter, clearer statements for better readability.

The authors' response:

Dear reviewer! The sentence was broken into shorter. (lines 59-62)

II. The connection between seasonal factors and specific mechanisms (vitamin D pathways, allergen exposure) could be included with more current evidence.

The authors' response:

Dear reviewer! We have added additional information to the Introduction section. (lines 62-67)

3. Methods: The manuscript does not address how missing data were managed. Authors should either: Describe the specific approach used for handling missing values (e.g., complete case analysis, multiple imputation) OR explicitly state if there were no missing values in the dataset

The authors' response:

Dear reviewer! We excluded all cases in which at least one of the study variables was missing, so there were no missing values in the dataset. We have added additional information to the Methods section. (lines 99-100)

4. Results: The relatively low asthma prevalence (4.8%) compared to global ISAAC estimates (11.8-13.8%) requires more discussion.

The authors' response:

Dear reviewer! Our study revealed that, compared with global trends, children residing in Moscow, Russia, presented high prevalences of AD (29.4%) and AR (23.2%), with a somewhat lower asthma prevalence of 4.84%. The differences we obtained may be associated with different methodologies for collecting clinical and anamnestic data, and different climatic, geographical, and socio-economic characteristics of the regions. We have added additional information to the Discussion section. (lines 241-243)

5. Discussion: Recommended additional discussion of potential prevention strategies based on findings

The authors' response:

Dear reviewer! Specifically, based on our data, a potential strategy for preventing allergic diseases in children in Moscow could be planning pregnancy with the birth of children in the spring-summer period between March and July to reduce the risk of AD and in the spring-autumn period between May and September to reduce the risk of AR, especially in cases of family allergy history. Regardless of the season of birth, important preventive measures include: breastfeeding, correcting and maintaining normal vitamin D levels in blood, moisturizing and maintaining the skin's barrier function, reducing the infectious load, introducing complementary foods in a timely manner, creating a supportive social and psychological environment, etc. Furthermore, the influence of seasonality on the risk of developing allergy should be considered when planning further epidemiological studies, interpreting the data obtained, and developing prevention strategies based on them. We have added additional information to the Discussion section. (lines 340-350)

6. Limitations: Missing discussion about generalizability beyond Moscow

The authors' response:

Dear reviewer! The sample included only children living in Moscow and did not include children living in other regions of Russia. We have added information about this limitation. (lines 376-378)

7. Conclusion: Recommended to include clinical implications discussion for prevention strategies

The authors' response:

Dear reviewer! Based on our data, an additional potential strategy for preventing allergic diseases among children living in Moscow could be planning pregnancy with the birth of children in the spring-summer period between March and July to reduce the risk of AD and in the spring-autumn period between May and September to reduce the risk of AR, especially in cases of family allergy history. Further studies are needed to gather evidence for the observed associations of season and month of birth with disease risk while considering additional factors and to better understand the mechanisms underlying this association. Furthermore, the possible association between season of birth and the risk of developing allergies should be taken into account when planning further epidemiological studies, interpreting the obtained data and developing preventive strategies based on them. We have added additional information to the Conclusion section. (lines 388-397)
